# Current and future suitable habitat areas for *Nasuella olivacea* (Gray, 1865) in Colombia and Ecuador and analysis of its distribution across different land uses

**DOI:** 10.3897/BDJ.8.e49164

**Published:** 2020-01-28

**Authors:** Pablo Medrano-Vizcaíno, Patricia Gutiérrez-Salazar

**Affiliations:** 1 School of Biological Sciences, University of Reading, Reading, United Kingdom School of Biological Sciences, University of Reading Reading United Kingdom; 2 Grupo de Investigación Ambiental para el Desarrollo Sustentable (GIADES), Universidad Politécnica Salesiana, Quito, Ecuador Grupo de Investigación Ambiental para el Desarrollo Sustentable (GIADES), Universidad Politécnica Salesiana Quito Ecuador

**Keywords:** Ecological Niche, Maxent, Tropical Andes, Mountain Coati, Procyonidae

## Abstract

*Nasuella olivacea* is an endemic mammal from the Andes of Ecuador and Colombia. Due to its rarity, aspects about its natural history, ecology and distribution patterns are not well known, therefore, research is needed to generate knowledge about this carnivore and a first step is studying suitable habitat areas. We performed Ecological Niche Models and applied future climate change scenarios (2.6 and 8.5 RCP) to determine the potential distribution of this mammal in Colombia and Ecuador, with current and future climate change conditions; furthermore, we analysed its distribution along several land covers. We found that *N. olivacea* is likely to be found in areas where no records have been reported previously; likewise, climate change conditions would increase suitable distribution areas. Concerning land cover, 73.4% of *N. olivacea* potential distribution was located outside Protected Areas (PA), 46.1% in Forests and 40.3% in Agricultural Lands. These findings highlight the need to further research understudied species, furthering our understanding about distribution trends and responses to changing climatic conditions, as well as informig future PA designing. These are essential tools for supporting wildlife conservation plans, being applicable for rare species whose biology and ecology remain unknown.

## Introduction

*Nasuella olivacea* (Gray, 1865) is a rare and small carnivore, endemic to the forests and paramo of the Andes of Colombia and Ecuador (Balaguera-Reina et al. 2009). Its altitudinal range varies from 1,300 to 3,862 m of elevation in Ecuador (Medrano-Vizcaíno 2018), but it gets up to 4,260 m in Colombia (Balaguera-Reina et al. 2009, Helgen et al. 2009). The climate where this species inhabits ranges from 9-24°C with an annual precipitation rate of 1,600–2,400 mm (Delgado-V. 2009, Sánchez et al. 2004).

In Ecuador, its presence is reported in the provinces of Imbabura, Carchi, Pichincha, Cotopaxi, Bolívar, Tungurahua, Chimborazo, Cañar, Azuay, Loja and Napo (Vallejo 2017), while in Colombia, it has been reported in 12 out of 32 departments (Balaguera-Reina et al. 2009, Ponce et al. 2016). Nevertheless, most of the records have been collected near Bogotá (Guzmán-Lenis 2004).

Although this species maintains a wide distribution range due to its tolerance to habitat alterations (González-Maya et al. 2016), it has been negatively affected by deforestation, hunting, agricultural expansion, social conflicts and attacks by domestic animals (Balaguera-Reina et al. 2009, Zapata-Ríos and Branch 2018). Consequently, according to the UICN, it is considered near threatened (NT) and it is estimated that only 36% of its distribution area is located in forests remnants (Helgen et al. 2009).

*Nasuella olivacea* is diurnal, terrestrial, arboreal and gregarious (only adult males are solitary; Vallejo 2017, Tirira 2017). Its diet is omnivorous, based on vegetables, fruits, vertebrates and invertebrates, showing preference for the consumption of Coleoptera, Orthoptera, Myriapoda and Hymenoptera insects, but adults show a wider trophic niche that include amphibians (Rodríguez-Bolaños et al. 2000).

In Colombia, *Nasuella olivacea* occurs in sympatry with *Nasua nasua* (González-Maya et al. 2015); nevertheless, it is not known how these two species can share and compete in the same habitat, but it is known that they have the same diet (Balaguera-Reina et al. 2009). Considering competence between both species, the possible niche overlap is a great disadvantage for the Mountain Coati, because its population density in Andean forests is almost 0.0035 ind/km, which is very small compared with *N. nasua* population density, which reaches 0.17 ind/km (Sánchez et al. 2008a). In Ecuador, no sympatric records with *Nasua nasua* have been reported. Indeed, research about this carnivore is scarce, with only two scientific documents available (Ramírez 2011, Medrano-Vizcaíno 2018) and some aspects about its biology are assumed to be similar to *Nasua nasua* (Linnaeus, 1766) (Tirira 2017).

In general, current information on distribution limits of most species in the tropical Andes is scarce (Buytaert et al. 2014, Vuille et al. 2003), but it is known that changes in global temperature would cause different distribution patterns ([Bibr B5452188][Bibr B5451496]) and species from mountain tropical ecosystems are more vulnerable because climate alterations are more remarkable at higher altitudes (Bradley 2006, Vuille et al. 2008). The Mountain Coati has gone through adaptative processes to live in forests and highlands (Rodríguez-Bolaños et al. 2000), but it is not known how climate change will influence its distribution.

A useful tool to understand these distribution processes is working with Ecological Niche Models (ENMs) as they provide predictions of suitable areas for species distribution (Lee‐Yaw et al. 2016) by analysing environmental/spatial variables together with occurrence records (Warren and Seifert 2011). Moreover, such models can also be applied to predict the effects of climate change on future species distributions (Searcy and Shaffer 2016); hence, these tools have become very important for ecological and conservation research (Guisan and Zimmermann 2000). One of the most used tools for modelling and mapping species distributions is MaxEnt, which generates an index of relative habitat suitability (Fitzpatrick et al. 2013).

This research determines potential areas where *N. olivacea* currently occurs and potential distribution areas under two different climate change scenarios. In addition, we analyse how this species is distributed along Protected Areas (PA) and different land covers.

## Material and methods

For the distribution analysis, we used data from Global Biodiversity Information (GBIF; www.gbif.org) and scientific literature where the presence of this mammal is reported (Brito and Ojala-Barbour 2016, Medrano-Vizcaíno 2018, Ramírez 2011, Zapata-Ríos and Branch 2018). We excluded repeated records using a 1 km^2^ cell size; therefore, one record per cell was validated. Later, we performed maximum entropy models with MaxEnt 3.4.1 (Phillips et al. 2006) using 19 climate variables with a resolution of 30 seconds (1 km^2^) obtained from WorldClim (Hijmans et al. 2005; http://www.worldclim.org/download) to identify potential distribution areas for *Nasuella olivacea* under current and future conditions (climate change scenarios). We filtered these variables to work with the most important and the least correlated ones. To select the most important variables, we ran a previous model with MaxEnt and analysed the jackknife test. Additionally, we eliminated high correlated variables (> 0.8) using the Pearson correlations matrix, which is a useful tool to avoid multicollinearity (Merow et al. 2013).

Using the filtered variables, we executed 100 runs of the model with the resample method of bootstrap, 30% of the records being used for validation of the models and 70% for its generation. For the models with climate change, we used projected variables for the year 2050 (average between 2041 and 2060). We applied the General Circulation Model (GCM) HadGEM2-ES (Martin et al. 2011) because it is considered stable, realistic and has good performance in the tropics (Collins et al. 2011, Jones et al. 2011, Martin et al. 2010). Moreover, we used Representative Concentration Pathways (RCP), which are climate change projections that consider different situations of greenhouse and CO2 emissions, social and economic aspects and climate change mitigation policies (Qin et al. 2016). Therefore, we applied two RCP scenarios: 1) RCP 2.6; it is the most optimistic scenario, climate change mitigation policies are strong, greenhouse and CO2 emissions are reduced and hence, it is the least climate change situation (Butler et al. 2012, Varela et al. 2015); and 2) RCP 8.5; an extreme climate change scenario, climate change mitigation policies do not exist and greenhouse and CO2 emissions are increasing (Ruosteenoja et al. 2016, Castillo et al. 2017, Varela et al. 2015). The assessment of the model was performed using the AUC value (Area Under the Curve-ROC). To evaluate possible variations in the potential distribution of this mammal, we measured the distribution areas with the three scenarios: current climate and future climate change scenarios RCP 2.6 and RCP 8.5.

Finally, we quantified how its presence is distributed along PA and several categories of land cover. For this, we used the generated potential distribution map with current climate conditions, PA shapefiles (Ministerio de Ambiente del Ecuador 2012, Parques Nacionales Naturales de Colombia 2015) and land cover shapefiles of Colombia and Ecuador (IGAC 2012, MAE-MAGAP 2015). To conduct analysis with land cover, we worked with the next categories: 1) Forests (primary forests, secondary forests, fragmented forests, gallery forests and forest plantations), 2) Agricultural lands (crops and pasturelands), 3) Shrubs and herbaceous vegetation, 4) Anthropic areas (Human settlements and infrastructures), 5) Near water bodies and 6) Other areas (rocky outcrops, glacial or nival areas, swamps, peat bogs and degraded lands). All the maps and geographical analysis were performed with the software QGIS (Quantum GIS Development Team 2018).

## Results

**Figure 1.**
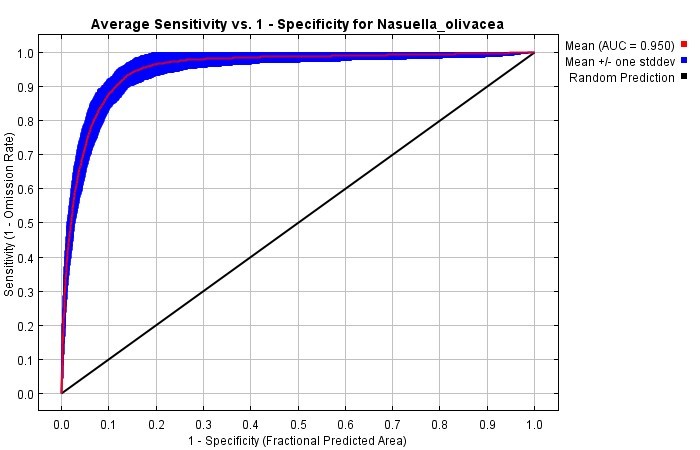
Model assessment using the Area Under the Curve (AUC).

We obtained 58 records of *N. olivacea*, from Ecuador and Colombia (Suppl. material 1) and 7 climate variables that were the least correlated and the most important for the models. The percentage of contribution of each variable was: BIO8 = Mean Temperature of Wettest Quarter (64%); Bio4 = Temperature Seasonality (standard deviation *100) (17.9%); Bio19 = Precipitation of Coldest Quarter (5.5%); Bio17 = Precipitation of Driest Quarter (4.4%); Bio18 = Precipitation of Warmest Quarter (3.2%); Bio7 = Temperature Annual Range (BIO5-BIO6) (3.1%); and Bio15 = Precipitation Seasonality (Coefficient of Variation) (1.9%). The AUC value of the model was 0.95, which means an excellent data adjustment (Fig. 1).

**Figure 2.**
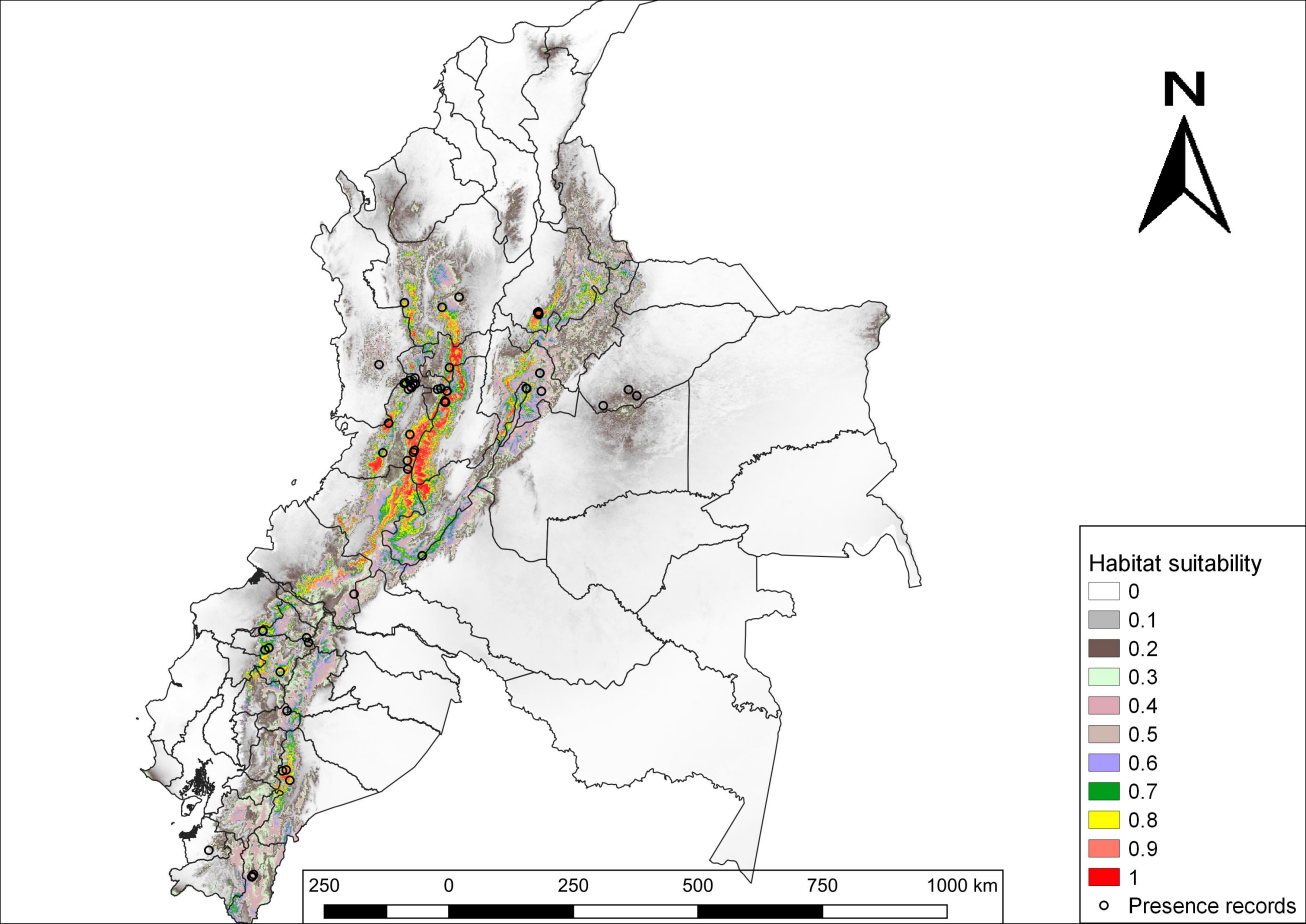
Current habitat suitability of *N. olivacea* in Ecuador and Colombia. The probabilities of habitat suitability vary from 0 (lowest habitat suitability) to 1 (highest habitat suitability).

In general, results obtained for the current potential distribution in Ecuador and Colombia reveal that areas with the highest habitat suitability are highlands (Fig. 2). The highest habitat suitability (0.9-1) for *N. olivacea* in Ecuador is located in the province of Morona Santiago, Morona canton, Parishes: Río Blanco, Zuña and Alshi, followed by the province of Pichincha, Quito canton, Parishes: Píntag, Manuel Conejo Astorga and Lloa; and the province of Santo Domingo de los Tsáchilas also shows a relatively high probability in Santo Domingo canton, Alluriquín parish. Additionally, there is also an important habitat suitability (0.8-0.89) in the provinces of Carchi, Imbabura, Esmeraldas, Cotopaxi and Tungurahua. On the other hand, it is observed that suitable areas for the distribution of this species is higher in Colombia than in Ecuador. The departments of Tolima, Valle del Cauca, Cauca and Caldas show extensive areas with high habitat suitability (0.9-1).

**Figure 3.**
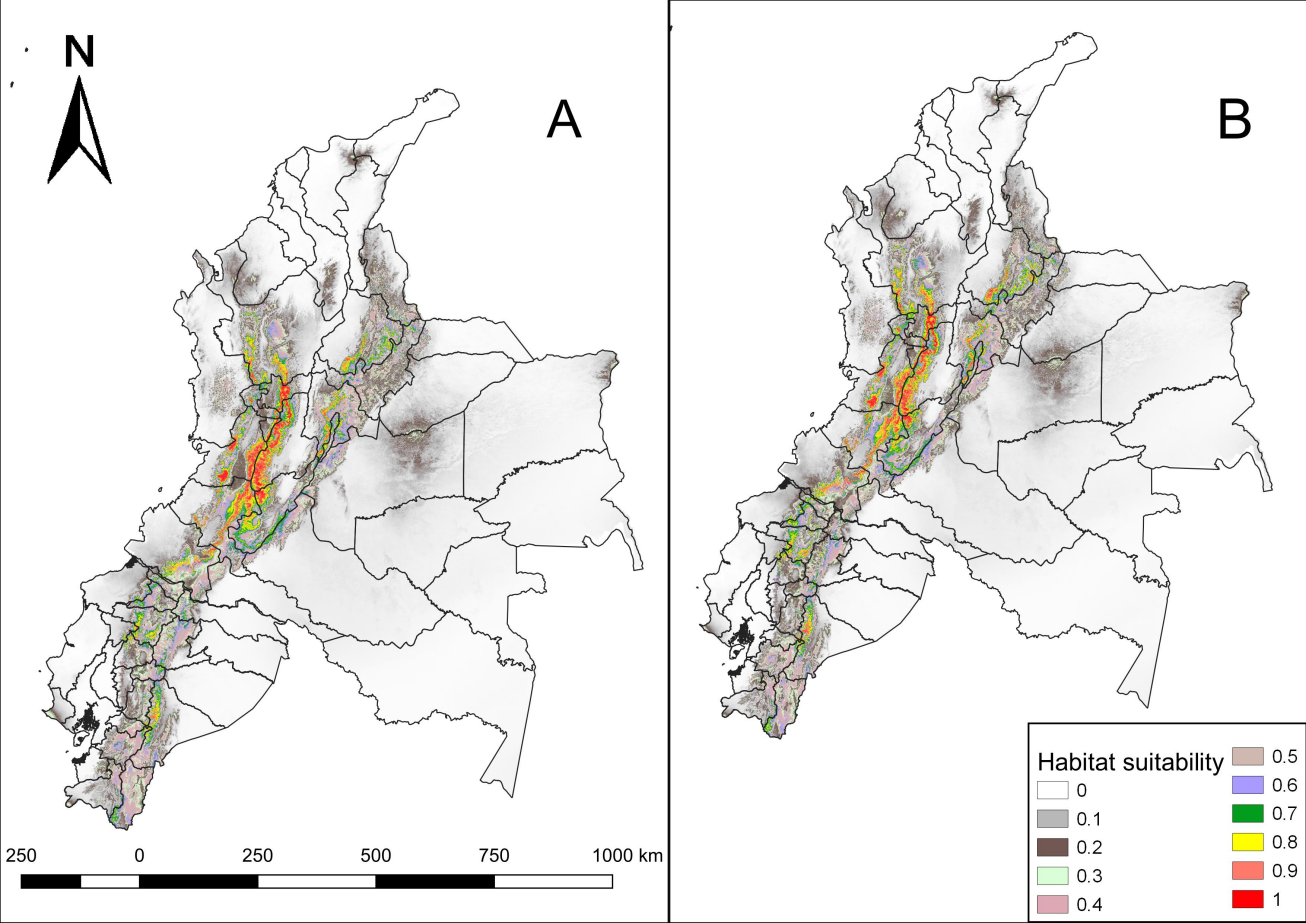
Future habitat suitability of *N. olivacea* in Ecuador and Colombia under climate change conditions. **A.** RCP; 2.6 **B.** RCP 8.5. The probabilities of habitat suitability vary from 0 (lowest habitat suitability) to 1 (highest habitat suitability).

Comparing the results, it is observed that the current potential distribution covers 93,190.26 km², the future model with RCP 2.6 scenario covers 99,231.7 km², while the future model with RCP 8.5 scenario covers 98,802.69 km² (Fig. 3).

**Figure 4.**
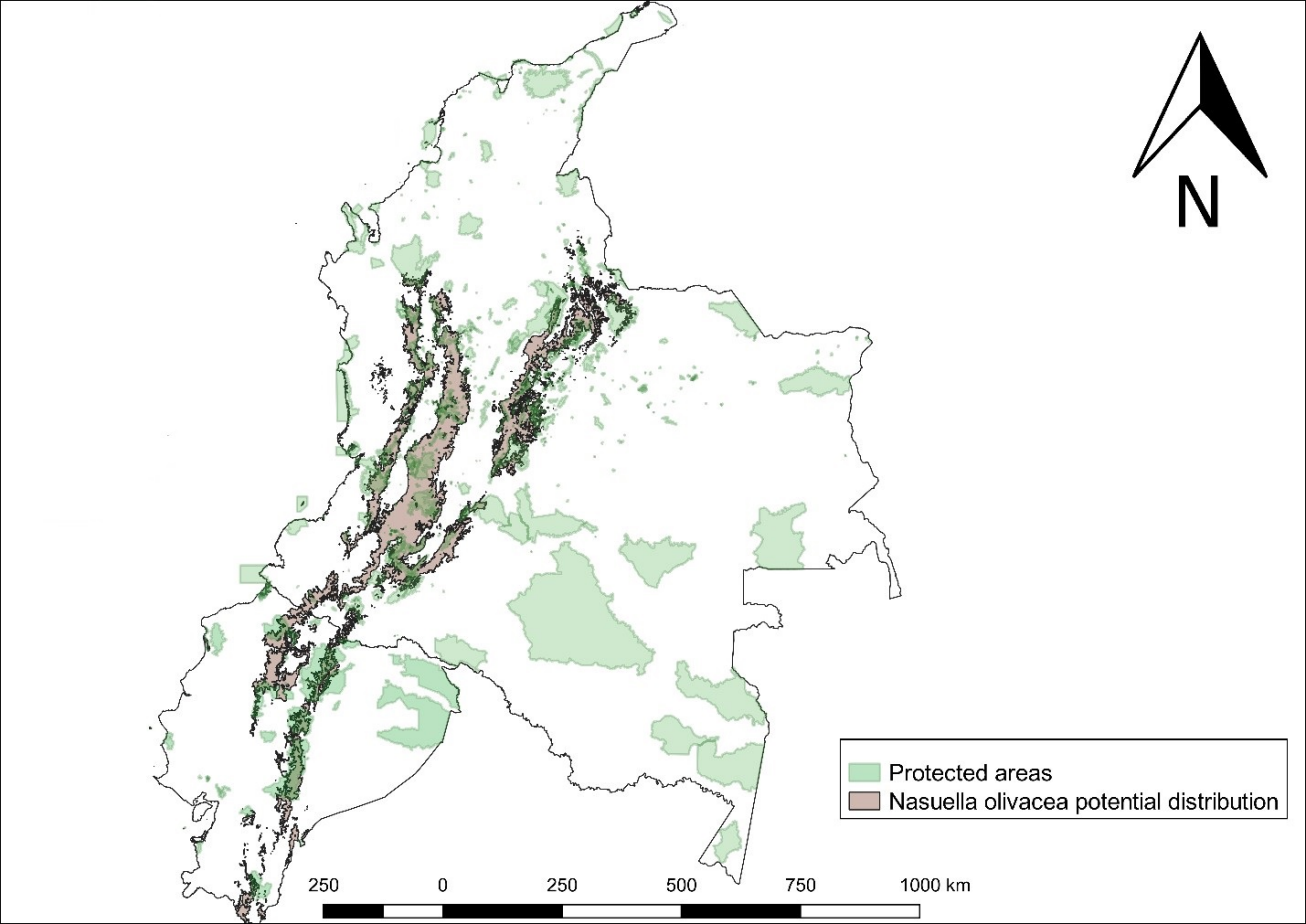
*Nasuella olivacea* potential distribution along PA in Colombia and Ecuador.

Regarding the factors that could threaten or favour the species population viability, we found that only 24,797.26 km² of its current potential distribution area (93,190.26 km²) are located inside PA. Then, most of its distribution is located outside PA (73.39%) (Fig. 4).

Analysing the results per country, in Colombia we have a potential distribution area of 73,082 km² and only 17,393.67 km² are inside PA; hence, 76.2% belongs to areas that do not have any protection figure, which is obviously negative for the conservation of the Coati. While in Ecuador, the potential distribution area is 20,108.26 km² and only 7,403.59 km² are inside PA; hence, 63.18% is found outside areas that could benefit its population viability (Table 1).

Regarding land cover, we found that *N. olivacea* is mainly distributed along forests and agricultural lands. There are different situations when this aspect is analysed independently for each country. In Ecuador, the distribution is mostly located along forests (60.82%), with a large differencecompared to Agricultural lands (23.47%); on the other hand, Colombia shows similar percentages for Forests (41.99%) and Agricultural lands (44.89%) (Table 2).

## Discussion

In Ecuador, our ENMs show high habitat suitability in the provinces of Morona Santiago, Santo Domingo and Esmeraldas; nevertheless, no field observations in published articles have been reported for these provinces. This finding is possibly explained because it is an understudied species in Ecuador; hence, distribution areas in this country could still not be well defined. However, it is also important to validate ENMs with fieldwork to avoid an overestimation of the predicted distribution areas (Contreras-Medina et al. 2010, Plasencia-Vázquez et al. 2014).

Another aspect to consider is that MaxEnt does not perform ENMs with natural history information (Buckley et al. 2010, Phillips et al. 2006); therefore, aspects like predation (Sánchez et al. 2008b, Hernández-Guzmán et al. 2010) and competence with other species (Balaguera-Reina et al. 2009) could limit potential distribution areas obtained with ENMs. Nevertheless, it has been useful to estimate the potential distribution of other understudied species with similar altitudinal ranges such as *Coendou rufescens*, where models were executed with fewer records than our study (Narváez-Romero et al. 2018).

According to our results, the variables BIO8 = Mean Temperature of Wettest Quarter, Bio4 = Temperature Seasonality and Bio19 = Precipitation of Coldest Quarter represent the highest contribution for the model, which could be related with *N. olivacea* diet. As this species mainly feeds on invertebrates (which are abundant in rainy seasons), it is possible that low temperatures and precipitation play an important role for its distribution patterns (Sánchez et al. 2008a, Rodríguez-Bolaños et al. 2000).

Concerning climate change, we found that future scenarios with RCP 2.6 and RCP 8.5 would increase habitat suitability for *N. olivacea*. Considering that this species is known to inhabit a wide variety of habitats (Sánchez and Alvear 2003) and that prior studies have reported sympatry with the lowland coati *Nasua nasua* (Arias-Alzate et al. 2016, González-Maya et al. 2015), it is possible that *N. olivacea* does not have a restricted altitudinal habitat.

According to prior research, it is expected that species with no restricted altitudinal habitats could increase their distribution area when temperatures increase (Freeman et al. 2018). Moreover, the response of humid biomes where this carnivore mainly inhabits (such as paramo and montane forests) (Balaguera-Reina et al. 2009) to climate change, is an upward displacement of their upper and lower limits (Tovar et al. 2013). Therefore, the predicted increase in the distribution area of *N. olivacea* under climate change scenarios could be related to its necessity in finding areas climatically more adequate for its survival.

Analysing PA and land cover, we found that, although 46% of the current potential distribution area is located inside forests, only 26% is located inside PA. Likewise, agricultural expansion is another challenge to be solved, with 40% of the current potential distribution area located along agricultural lands. These results represent a great threat for the survival of this mammal, as a poultry predator and a plague for potato crops (Sánchez et al. 2008a), it is vulnerable to persecution from humans or domestic animals; moreover, some people hunt this animal to obtain its fur (Alroy 2001).

Forest recovery could represent an effective strategy for *N. olivacea* conservation, but it has to be conducted together with expanding PA, which have been also shown to be a good strategy for conservation and long term management of species (Cuesta et al. 2017). In the period 2001–2014, at 1,500–4,000 m, a ligneous vegetation gain of 130,000 and 190,000 ha has occurred in Ecuador and Colombia, respectively (Aide et al. 2019), which is a hopeful factor for the conservation of the Mountain Coati.

This article highlights some of the main threats that *N. olivacea* faces for its conservation; nevertheless, our results show that there is a lot to be known and to be done. Considering that this is the least studied carnivore in the world (Helgen et al. 2009), generating new knowledge is necessary to establish more effective conservation programmes. Determining habitat suitability areas for rare species is required to conduct new research. Therefore, we hope that our results work as a basis for more studies, which will be necessary to clarify unknown aspects of this mammal.

## Conclusions

Although future climate change scenarios (even the most pessimistic) would slightly increase the habitat suitability areas for the distribution of *Nasuella olivacea*, agricultural activities appear as a potential threat for this species. In addition, our results suggest that PA are not playing an important role for the conservation of this carnivore, which would mean that conservation strategies in Ecuador and Colombia need to be reinforced to protect this species.

## Supplementary Material

99B1AAAF-BA5C-5226-A867-C4D15F042FFA 10.3897/BDJ.8.e49164.suppl1 Supplementary material 1 Records of *Nasuella olivacea* in Colombia and Ecuador Data type: Occurrences
Brief description: Records used for modelling the potential distribution of *Nasuella olivacea* in Colombia and Ecuador
File: oo_371412.csv https://binary.pensoft.net/file/371412 Pablo Medrano-Vizcaíno, Patricia Gutiérrez-Salazar

## Figures and Tables

**Table 1.** Potential distribution along PA in Ecuador and Colombia.

**Protected areas (PA)**	**Colombia (km²)**	%	**Ecuador (km²)**	%	**Total**	%
**Inside PA**	17,393.67	23.8	7,403.59	36.82	24,797.26	26.61
**Outside PA**	55,688.33	76.2	12,704.67	63.18	68,393	73.39
**Total**	73,082	100	20,108.26	100	93,190.26	100

**Table 2.** Potential distribution area along different land cover categories.

**Land cover**	**Colombia (km²)**	%	**Ecuador (km²)**	%	**Total**	%
Forests	30,689.12	41.99	12,230.23	60.82	42,919.35	46.05
Agricultural lands	32,808	44.89	4,720	23.47	37,528	40.27
Shrubs and herbaceous vegetation	8,425.25	11.53	2,630.4	13.08	11,055.65	11.86
Anthropic areas	502.27	0.69	178.43	0.89	680.7	0.73
Near water bodies	77.73	0.11	63.33	0.31	141.06	0.15
Other areas	579.63	0.79	285.87	1.42	865.5	0.93
**Total**	**73,082**	**100**	**20,108.26**	**100**	**93,190.26**	**100**
